# Primary bladder amyloidosis mimicking bladder cancer complicated by bladder rupture: A case report

**DOI:** 10.1002/ccr3.5140

**Published:** 2021-11-22

**Authors:** Rayan M. Sibira, Ahmed Albakar, Nagy Younes, Issam A. Albozom, Khalid Al Rumaihi

**Affiliations:** ^1^ Department of Laboratory Medicine and Pathology Hamad Medical Corporation Doha Qatar; ^2^ Urology Department Hamad Medical Corporation Doha Qatar

**Keywords:** amyloidosis, bladder cancer, hematuria, transurethral resection

## Abstract

Amyloidosis is related to the extracellular deposition of abnormal protein fibrils in various tissues. It can be either localized to an organ or generalized, affecting multiple systems. Amyloidosis of the urinary bladder is a rare histopathological finding. It is clinically interesting that such cases' clinical, radiological, and even endoscopic presentation mimic urothelial carcinoma to a great extent. Here, we discuss a case of a 49‐year‐old gentleman who presented with frank painless hematuria. The patient was diagnosed with a bladder mass suspicious of malignancy depending on the clinical presentation aided by the cystoscopic and radiological evaluation. Histopathologic samples of the transurethral resection of the mass proved to be primary bladder amyloidosis. This case is of unique clinical interest in that it is the first case reported of bladder amyloidosis that is complicated by extraperitoneal bladder rupture post‐operatively. However, no immediate intraoperative perforation to the bladder wall during resection was evidenced.

## Graphical abstract

Primary urinary bladder amyloidosis is a rare histopathological finding. Interestingly, it presents clinically and radiologically similar to urothelial carcinoma of the bladder. The only differentiation can be reached by histopathologic examination. Resection of such bladder lesions requires special care as such flat pathological lesions can be complicated by bladder perforation.

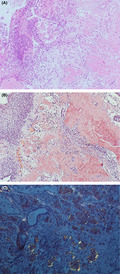

## 1 INTRODUCTION

Amyloidosis refers to a non‐neoplastic heterogeneous group of disorders related to the extracellular deposition of insoluble fibrils in different organs.^1^ Amyloidosis can be primary, secondary, and hereditary. Primary amyloidosis (AL) is usually related to plasma cell abnormalities. On the contrary, secondary amyloidosis (AA) occurs due to long‐standing infections, inflammation, or neoplastic insults. Eventually, hereditary amyloidosis (ATTR) is related to autosomal‐dominant inherited mutations in transthyretin protein gene.^1^ Amyloidosis can be systemic, which is usually progressive and fatal or localized. Localized amyloidosis of the urinary bladder is rare, with the most extensive series reported 31 cases.^2^ The clinical importance of bladder amyloidosis is that it mimics bladder cancer. Although amyloidosis is a benign condition, it was associated with urothelial carcinoma in 48% of 21 cases reported.^3^ Here, we present a case of localized bladder amyloidosis with a review of the literature. Interestingly, this is the first case reported in which resection of this bladder lesion was complicated by postoperative extraperitoneal perforation of the bladder wall.

## 2 CASE PRESENTATION

**FIGURE 1:**
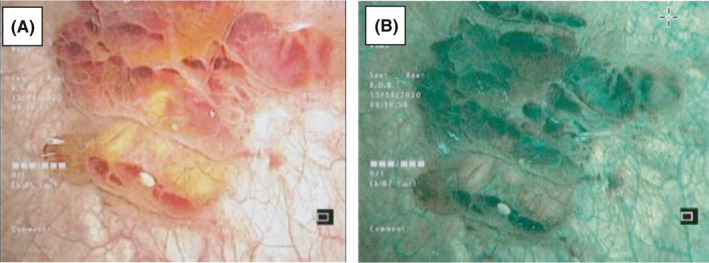
(A) Flexible cystoscopy of bladder lesion. (B) Narrow band imaging (NBI) cystoscopy of the bladder lesion

A 49‐year‐old Indian gentleman presented to our clinic complaining of recurrent attacks of painless frank terminal hematuria for two months. This picture was associated with frequency, urgency, and nocturia three times per night. On the contrary, there was no history of urinary tract infections or stone disease. He was a non‐smoker, and we could not identify any family history of malignancy. On examination, the patient had mild suprapubic tenderness, otherwise no abnormal findings. Urine microscopy showed 3 white blood cells (WBCs)/high‐power field (HPF) and 206 red blood cells (RBCs)/high‐power field (HPF). Urine cytology showed atypical urothelial cells. At the first clinic visit, flexible cystoscopy revealed a cystic hyperemic growth at the left lateral bladder wall (Figure 1A‐B). Computed tomography (CT) scan with contrast was done two days later, showing focal bladder wall thickening related to the left lateral bladder wall suspicious for either an inflammatory or a malignant condition. The patient was booked for transurethral resection of the bladder tumor (TURBT).

After six weeks from the CT scan, the patient was admitted for surgery. Intraoperatively, the lesion was seen on the left lateral bladder wall and was resected using the monopolar transurethral resectoscope. No immediate surgical complication was seen intraoperatively. The bladder wall was intact with clear urine outflow. No postoperative intravesical therapy was given as the surgical team preferred to assess the histopathological diagnosis first. A foley's catheter was inserted after the operation. The patient was discharged on the same day after catheter removal and could void clear urine smoothly.

**FIGURE 2:**
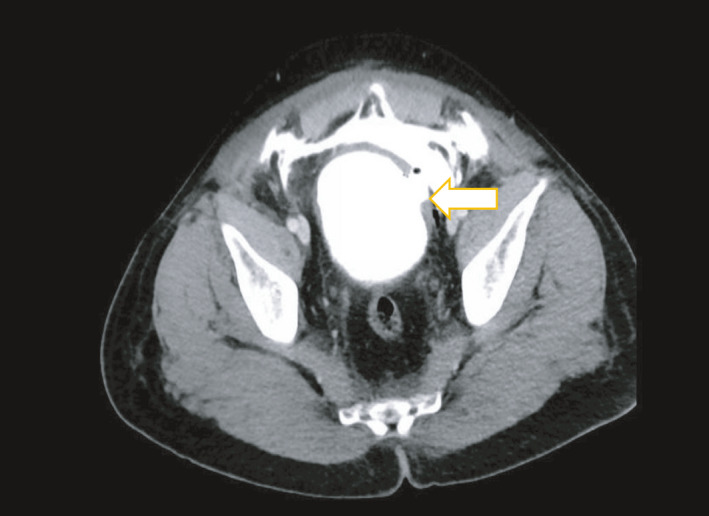
Abdominopelvic CT scan showed extravasation of contrast from the bladder to the pelvis through left lateral bladder wall defect

Two days after discharge, the patient presented to the emergency department complaining of hematuria, nausea, vomiting, generalized weakness, and lower abdominal pain for two days. Physical examination showed stable vital signs with moderate lower abdominal tenderness. Blood tests showed leukocytosis with WBCs of 16 × 1,000/Ul, a drop of hemoglobin by 2 g, and hyponatremia. Foley's catheter was reinserted. CT abdomen and pelvis (Figure 2) showed extravasation of contrast from the bladder to the pelvis through a 2 cm bladder wall defect related to the left lateral bladder wall. A diagnosis of extraperitoneal bladder rupture was established. Foley's catheter was kept on free drainage for seven days. The catheter was removed in the clinic follow‐up after a cystogram that showed no extravasation of contrast.

## 3 PATHOLOGICAL FINDINGS

**FIGURE 3:**
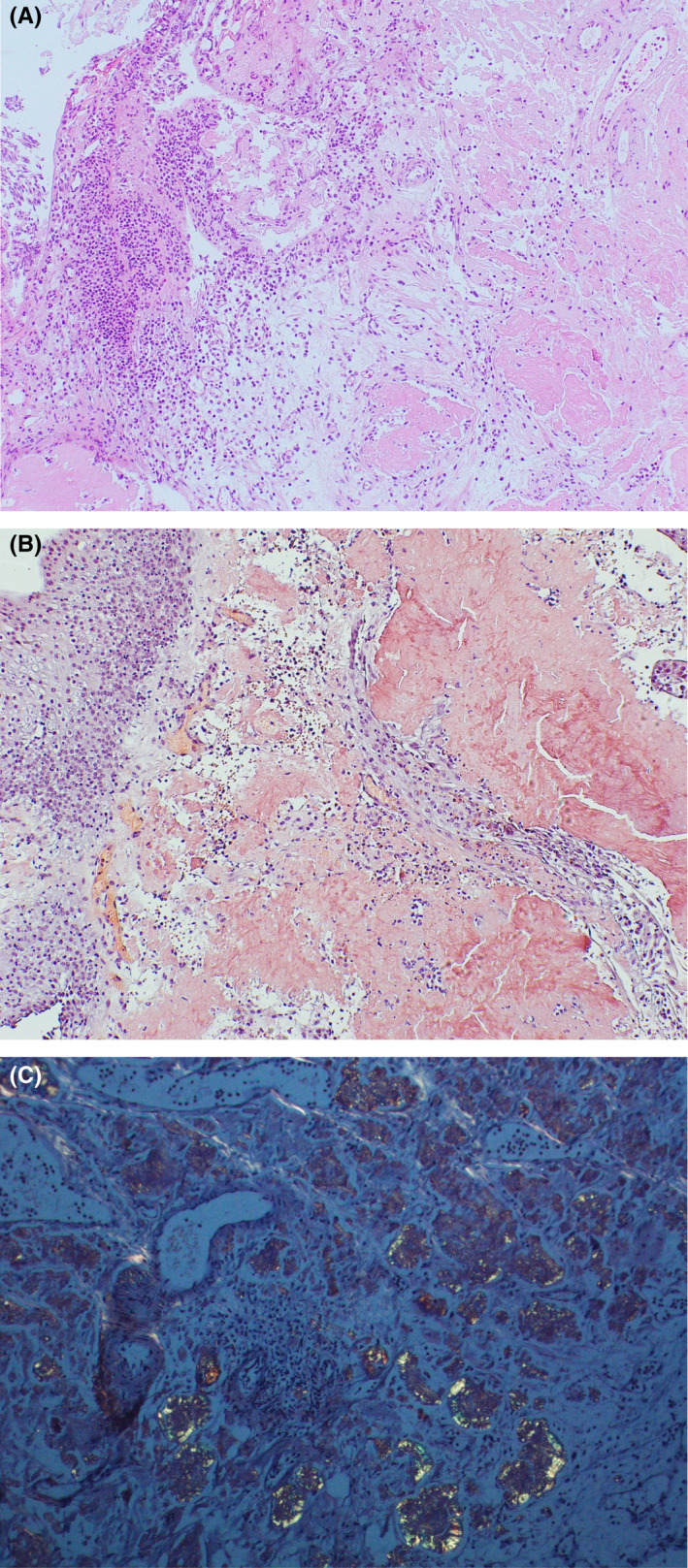
Urinary bladder amyloidosis. (A) A higher magnification of amyloid material (×200). (B) Congo red special stain with salmon‐pink color (×200). (C) Congo red special stain with apple‐green birefringence under polarized light microscopy (×200)

Microscopic examination (Figure 3A‐C) revealed several mucosal fragments of urothelial mucosa with underlying lamina propria significantly expanded by amorphous deposits of paucicellular, pale pink, homogenous proteinaceous material (Figure 3A), in a background of acute inflammation. This material by congo red special stain (Figure 3B) appeared deeply orangeophilic (salmon‐pink color) and demonstrated apple‐green birefringence (Figure 3C) under the polarizing lens. Immunohistochemical stains were positive for amyloid P protein stain.

Patient was referred to hematologist for evaluation after the diagnosis of primary amyloidosis was established. Workup revealed normal serum kappa‐ and lambda‐free light chains, and normal kappa/lambda‐free light chain ratio. Serum protein electrophoresis was essentially normal, and no monoclonal band was detected. Though primary localized (bladder) amyloidosis is the favorable diagnosis, patient was followed for two months postoperative and was asymptomatic.

## 4 DISCUSSION

*Amyloidosis* is a rare disease that occurs when an abnormal protein (amyloid) builds up in organs and interferes with their normal function. Amyloidosis is divided into localized and systemic subtypes. Localized amyloidosis affects only one body organ or tissue type (eg, amyloid β associated with Alzheimer's disease, AIAPP (amylin) associated with type 2 diabetes, and Acal (calcitonin) associated with medullary thyroid cancer).^4^ Systemic amyloidosis affects more than one body organ or system. It is subcategorized into primary, secondary, dialysis‐related, and hereditary subtypes. In primary amyloidosis (most common subtype), amyloid light chain and amyloid heavy chain, derived from immunoglobulins light and heavy chains, as seen in plasma cell disorders.^4^ Secondary amyloidosis, AA, derived from serum amyloid A protein, an acute‐phase reactant produced by the liver,^5^ always occurs as a complication of an underlying chronic inflammatory process. Dialysis‐related amyloidosis, beta2‐microglobulin accumulates in the bone, periarticular structures, and viscera of patients with chronic kidney disease. Hereditary amyloidosis occurs with mutations in the TTR gene (TTRv, variant) and is associated with cardiac failure and polyneuropathy.^4^

Primary localized amyloidosis of the urinary bladder is a rare disorder that is clinically and radiographically difficult to distinguish from urothelial carcinoma like in this case. Depending on the degree of disease involvement, patients may present with painless hematuria, irritative urinary symptoms, and obstructive symptoms.^6^ Ultrasound, CT scan, and MRI are often used as initial methods to evaluate patients with urinary bladder amyloidosis. MRI T2‐weighted imaging can distinguish it from urothelial carcinoma by characteristic hypointense lesion with noticeable mass effect.^7^ Urine cytology cannot distinguish amyloidosis from carcinoma because of the subendothelial location of most amyloid deposits and limited sensitivity for urothelial carcinoma.^8^ Definitive diagnosis of amyloidosis is achieved with biopsy. Like in our case, congo red staining of amyloid under light microscopy with polarized light produced the appearance of apple‐green birefringence. Immunohistochemistry for amyloid P protein confirms the diagnosis of amyloidosis.

Although amyloidosis and bladder cancer's gross picture can look similar, it is easy to differentiate amyloidosis from urothelial carcinoma by light microscopy. On the one hand, amyloidosis is an acellular process, but on the other hand, urothelial carcinoma is a very cellular process. Another differential diagnosis besides urothelial carcinoma is chronic cystitis with fibrosis. Amyloidosis and cystitis can be associated with severe inflammation and deposition of proteinaceous material. Profoundly, the proteinaceous material in amyloidosis has a characteristic dense pink homogeneous appearance by H and E, a deeply orangeophilic color by congo red, and apple‐green birefringence when polarized by a polarized lens.

## 5 CONCLUSION

To our knowledge, primary urinary bladder amyloidosis is an unusual disease. Our findings indicate that urinary bladder is a potential site for primary localized amyloidosis. A diagnosis of bladder amyloidosis is challenging, particularly when the primary bladder malignancy has been suspected. It is important for practicing surgical pathologists and urologist to be aware of the unusual findings of urinary bladder amyloidosis for accurate diagnosis and workup.

## CONFLICT OF INTEREST

The authors declare that they have no conflict of interest.

## AUTHOR CONTRIBUTIONS

Dr. Rayan M. Sibira, Dr. Ahmed Albakar, and Dr. Issam A. Albozom designed the study, performed the experimental work, analyzed and interpreted the data, prepared the manuscript, and critically reviewed the manuscript. Dr. Nagy Younes designed the study, performed the experimental work, prepared the manuscript, and critically reviewed the manuscript. Dr. Khalid Al Rumaihi critically reviewed the manuscript. All authors read and approved the final manuscript, and agreed on submission.

## CONSENT

Written informed consent was obtained from the patient to publish this report in accordance with the journal's patient consent policy.

## Data Availability

We are disclosing that strictest confidence was maintained for data collection as well as access and application in the study. Data were never shared at any level with any individuals not authorized to access research material. Data were only available upon request by the authors following permission from ABHATH Medical Research Center at Hamad Medical Corporation, Doha, Qatar. We fully understand that the use of confidential data for personal purposes is prohibited.

## References

[1] Baker KR , Rice L . The amyloidoses: clinical features, diagnosis and treatment. Methodist Debakey Cardiovasc J. 2012;8(3):3‐7. 10.14797/mdcj-8-3-3 PMC3487569 23227278

[2] Tirzaman O , Wahner‐Roedler DL , Malek RS , Sebo TJ , Li CY , Kyle RA . Primary localized amyloidosis of the urinary bladder: a case series of 31 patients. Mayo Clin Proc. 2000;75(12):1264‐1268. 11126834 10.4065/75.12.1264

[3] Sirohi D , Gandhi J , Amin MB , Luthringer DJ . Amyloidosis of the bladder and association with urothelial carcinoma: report of 29 cases. Hum Pathol. 2019;93:48‐53. 31425694 10.1016/j.humpath.2019.08.011

[4] Picken MM . The pathology of amyloidosis in classification: a review. Acta Haematol. 2020;143(4):322‐334. 32392555 10.1159/000506696

[5] Westermark GT , Fandrich M , Westermark P . AA amyloidosis: pathogenesis and targeted therapy. Annu Rev Pathol. 2015;10:321‐344. 25387054 10.1146/annurev-pathol-020712-163913

[6] DeSouza MA , Rekhi B , Thyavihally YB , Tongaonkar HB , Desai SB . Localized amyloidosis of the urinary bladder, clinically masquerading as bladder cancer. Indian J Pathol Microbiol. 2008;51(3):415‐417. 18723977 10.4103/0377-4929.42547

[7] Kato H , Toei H , Furuse M , Suzuki K , Hironaka M , Saito K . Primary localized amyloidosis of the urinary bladder. Eur Radiol. 2003;13(suppl 6):L109‐L112. 10.1007/s00330-002-1793-4 16440218

[8] Zhou F , Lee P , Zhou M , Melamed J , Deng FM . Primary localized amyloidosis of the urinary tract frequently mimics neoplasia: a clinicopathologic analysis of 11 cases. Am J Clin Exp Urol. 2014;2(1):71‐75. 25374907 PMC4219293

